# The Impact of White Blood Cell Count Trajectories on Prognosis and Secondary Acute Kidney Injury in Sepsis Patients

**DOI:** 10.1155/mi/6750509

**Published:** 2026-02-13

**Authors:** Jie Yan, Ailifeire Abudurexiti, Guligeina Yibubula, Wencai Li, Jing Liang

**Affiliations:** ^1^ Clinical Laboratory Diagnostics, The Sixth Affiliated Hospital of Xinjiang Medical University, Urumqi, 830002, Xinjiang, China, xjmu.edu.cn; ^2^ Sixth Clinical Medical College, Xinjiang Medical University, Urumqi, 830002, Xinjiang, China, xjmu.edu.cn; ^3^ Neurology, The Sixth Affiliated Hospital of Xinjiang Medical University, Urumqi, 830002, Xinjiang, China, xjmu.edu.cn; ^4^ General Practice, The Sixth Affiliated Hospital of Xinjiang Medical University, Urumqi, 830002, Xinjiang, China, xjmu.edu.cn; ^5^ Department of Blood Transfusion, The Sixth Affiliated Hospital of Xinjiang Medical University, No. 39 Wuxing South Road, Urumqi, 830002, Xinjiang, China, xjmu.edu.cn

**Keywords:** acute kidney injury, group-based trajectory modeling, mortality, sepsis, white blood cell trajectory

## Abstract

**Background:**

Sepsis‐associated acute kidney injury (SA‐AKI) is a common and severe complication in critically ill patients, yet the prognostic value of longitudinal white blood cell (WBC) dynamics remains underexplored. Most studies rely on single‐timepoint measurements, potentially overlooking important dynamic information for risk stratification.

**Objectives:**

This study aimed to identify distinct WBC trajectory patterns during the first 7 days of ICU admission and evaluate their associations with mortality and secondary AKI in sepsis patients.

**Methods:**

This retrospective cohort study analyzed 15,328 adult sepsis patients from the MIMIC‐IV database (version 3.1) between 2008 and 2019. Group‐based trajectory modeling (GBTM) was applied to daily WBC counts to identify trajectory subgroups. The primary outcome was 28‐day mortality, with secondary outcomes including 90‐day mortality and secondary AKI occurring after day 7. Cox proportional hazards regression was used to estimate hazard ratios with 95% confidence intervals. Subgroup analyses evaluated effect consistency across clinically relevant characteristics.

**Results:**

Four distinct WBC trajectory groups were identified: high (*n* = 812, 5.3%), medium–high (*n* = 2,830, 18.5%), medium–low (*n* = 7,121, 46.5%), and low (*n* = 4,565, 29.8%), with overall mean WBC counts of 24.13, 16.60, 11.24, and 6.64 × 10^9^/L, respectively. The 28‐day mortality rates were 31.2%, 20.7%, 14.8%, and 12.9% for high, medium–high, medium–low, and low groups, respectively (log‐rank *p*  < 0.001). Compared with the high group, the low trajectory demonstrated significantly reduced mortality risk (HR: 0.36, 95% CI: 0.31–0.42, *p*  < 0.001). The high‐trajectory group exhibited higher secondary AKI incidence (21.8% vs. 13.0%, *p*  < 0.001) and greater disease severity. A significant interaction was observed for AKI status (*P* for interaction = 0.003).

**Conclusions:**

Longitudinal WBC trajectory patterns provide superior prognostic information compared to single‐timepoint measurements in sepsis patients, with persistently high WBC levels associated with increased mortality and secondary AKI risk.

## 1. Introduction

Sepsis, defined as life‐threatening organ dysfunction caused by a dysregulated host response to infection, remains one of the most challenging syndromes in critical care medicine, affecting ~50 million patients annually worldwide and contributing to nearly 20% of all global deaths [1]. Among the spectrum of sepsis‐induced organ dysfunction, acute kidney injury (AKI) is particularly common, occurring in up to 62% of sepsis patients admitted to intensive care units and significantly amplifying morbidity and mortality risks [2]. Sepsis‐associated acute kidney injury (SA‐AKI) is not merely a marker of disease severity but an independent predictor of adverse outcomes, with affected patients demonstrating mortality rates approaching 48% and substantial risk of progression to chronic kidney disease [3]. The clinical burden of SA‐AKI underscores the urgent need for improved risk stratification tools to enable early identification of high‐risk patients and guide personalized therapeutic interventions.

The white blood cell (WBC) count has long been recognized as a fundamental biomarker in sepsis, reflecting the magnitude of systemic inflammatory response and immune activation [4]. Recent investigations have demonstrated that WBC‐related parameters, including neutrophil‐to‐lymphocyte ratio and other derived indices, possess significant prognostic value for predicting AKI and mortality in critically ill populations [5]. Furthermore, trajectory‐based analyses utilizing latent class modeling have emerged as powerful approaches to capture the dynamic nature of biomarker changes over time, revealing clinically meaningful subphenotypes with distinct outcomes [6]. Notably, a recent study identified eight distinct leukocyte trajectory subtypes in sepsis patients, with persistently high WBC levels associated with the poorest prognosis (HR: 3.00; 95% CI: 2.48–3.62) [7].

However, current understanding of WBC trajectories in sepsis remains limited by several factors. Most studies have focused on static, single‐timepoint measurements rather than longitudinal patterns, potentially overlooking important dynamic information [8]. Additionally, the relationship between WBC trajectory phenotypes and the development of secondary AKI beyond the initial resuscitation phase has not been systematically investigated. The clinical heterogeneity inherent to sepsis further complicates prognostication, as different patient subgroups may exhibit varying associations between inflammatory trajectories and outcomes [9]. These knowledge gaps limit the clinical utility of WBC monitoring for risk stratification and personalized management.

Therefore, this study aimed to identify distinct WBC trajectory patterns during the first 7 days of ICU admission using group‐based trajectory modeling (GBTM) in a large cohort of sepsis patients from the MIMIC‐IV database. We sought to characterize the clinical features associated with each trajectory subtype, evaluate their associations with 28‐ and 90‐day mortality, and assess the relationship between WBC trajectories and secondary AKI. Furthermore, we conducted subgroup analyses to examine the consistency of these associations across clinically relevant patient characteristics.

## 2. Materials and Methods

### 2.1. Study Design and Data Source

This retrospective cohort study utilized data from the Medical Information Mart for Intensive Care IV (MIMIC‐IV) database, version 3.1 (https://physionet.org/content/mimiciv/3.1/), which contains de‐identified electronic health records of patients admitted to the Beth Israel Deaconess Medical Center between 2008 and 2019 [10]. The database was accessed through PhysioNet after completion of the required training course and signing of the data use agreement. This study was conducted in accordance with the principles of the Declaration of Helsinki and followed the Strengthening the Reporting of Observational Studies in Epidemiology (STROBE) guidelines for observational research.

### 2.2. Study Population

**Figure 1:**
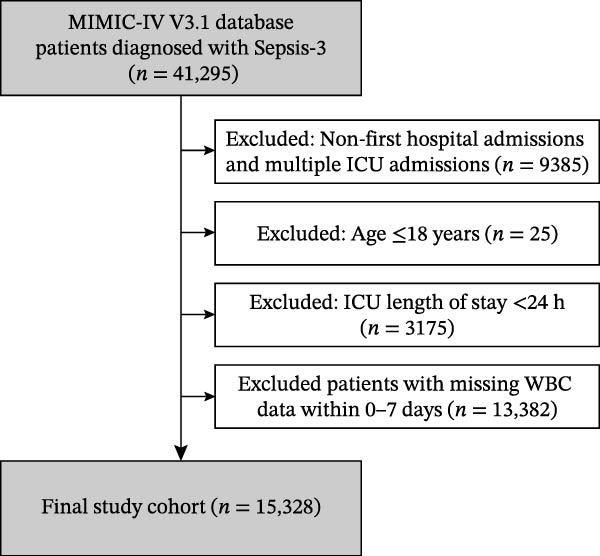
Flowchart of patient selection. A total of 41,295 patients diagnosed with Sepsis‐3 were identified in the MIMIC‐IV version 3.1 database. After applying exclusion criteria, 15,328 patients were included in the final study cohort.

Adult patients meeting the Sepsis‐3.0 diagnostic criteria were eligible for inclusion. Sepsis‐3.0 was defined as suspected infection with a Sequential Organ Failure Assessment (SOFA) score increase of two or more points from baseline. Exclusion criteria were as follows: non‐first hospital admissions or multiple ICU admissions during the same hospitalization, age 18 years or younger, ICU length of stay less than 24 h, and missing WBC count data within the first 7 days of ICU admission. The patient selection process is illustrated in Figure 1.

### 2.3. Data Extraction

Demographic characteristics including age, sex, and body mass index, were extracted at ICU admission. Disease severity was assessed using multiple scoring systems: SOFA score, Acute Physiology Score III (APS III), Simplified Acute Physiology Score II (SAPS II), Oxford Acute Severity of Illness Score (OASIS), Logistic Organ Dysfunction System (LODS) score, Glasgow Coma Scale (GCS), and Charlson Comorbidity Index. Clinical interventions within the first 24 h of ICU admission were documented, including mechanical ventilation, renal replacement therapy, coronary artery bypass grafting, percutaneous coronary intervention, intra‐aortic balloon pump placement, and hemodynamic monitoring devices. Medication use was recorded for vasopressors, sedatives, and insulin. Laboratory parameters and vital signs were collected daily from day 0 to day 7 following ICU admission. Daily WBC counts were extracted as the first available value within each 24 h period. When multiple measurements were obtained within the same 24 h interval, the average value was calculated. Our analysis was restricted to complete 24 h datasets, and any 24 h intervals containing missing values were omitted to ensure complete longitudinal data for trajectory modeling. Initial values within the first 24 h were used for baseline characterization.

### 2.4. Outcome Definitions

The primary outcome was 28‐day all‐cause mortality. The secondary outcome was 90‐day mortality. Secondary AKI was defined according to the Kidney Disease: Improving Global Outcomes (KDIGO) criteria, specifically identified as new‐onset AKI occurring after day 7 of ICU admission through hospital discharge, based on serum creatinine elevation of 0.3 mg/dL or greater within 48 h or an increase to 1.5 times baseline or greater within the preceding 7 days.

### 2.5. WBC Trajectory Analysis Using GBTM

GBTM was employed to identify distinct longitudinal patterns of WBC counts during the first 7 days of ICU admission. GBTM is a specialized application of finite mixture modeling that identifies clusters of individuals following similar developmental trajectories over time. The analysis was performed using the lcmm package in R software. Quadratic polynomial functions were specified for trajectory shapes to capture potential nonlinear patterns. The optimal number of trajectory groups was determined as four based on model fit statistics, clinical interpretability, and adequate sample sizes within each group. A grid search algorithm with 100 random starting values was implemented to ensure convergence to the global maximum likelihood solution. Patients were assigned to trajectory groups based on their highest posterior probability of group membership.

### 2.6. Trajectory Group Characterization and Nomenclature

Trajectory groups were characterized and named according to their mean WBC count levels across the observation period. Groups were designated as high, medium–high, medium–low, and low based on descending order of overall mean WBC values. The specific mean WBC counts at each time point for each trajectory group were calculated and reported to enable clinical interpretation and comparison with prior studies. This nomenclature was applied consistently throughout all subsequent analyses to facilitate clinical interpretation.

### 2.7. Clustering Quality Assessment

The quality of trajectory classification was evaluated using multiple complementary metrics. The Silhouette coefficient, ranging from negative one to positive one, assessed how well patients fit within their assigned groups compared to other groups. The Calinski–Harabasz index measured between‐cluster variance relative to within‐cluster variance. The Davies–Bouldin index quantified average similarity between each cluster and its most similar cluster, with lower values indicating better separation. Group balance was evaluated using entropy‐based measures to ensure adequate representation across trajectory groups.

### 2.8. Statistical Analysis

Baseline characteristics were compared across trajectory groups and between 28‐day survivors and non‐survivors. Continuous variables were presented as median with interquartile range due to non‐normal distributions confirmed by Shapiro–Wilk tests, and compared using Kruskal–Wallis tests. Categorical variables were presented as frequencies with percentages and compared using chi‐squared tests or Fisher’s exact tests as appropriate. Kaplan–Meier survival curves were constructed to visualize mortality differences across trajectory groups, with log‐rank tests for overall comparison. Cox proportional hazards regression models were used to estimate hazard ratios with 95% confidence intervals, using the trajectory group with the highest mortality rate as the reference category. Models were adjusted for clinically relevant covariates, including age, sex, body mass index, and disease severity scores.

### 2.9. Subgroup Analysis

Pre‐specified subgroup analyses were performed to assess the consistency of trajectory group associations with 28‐day mortality across clinically relevant patient subgroups. Subgroups were defined by sex, age (less than 65 years versus 65 years or older), body mass index categories, SOFA score (above versus below median), Charlson Comorbidity Index (above versus below median), mechanical ventilation use, vasopressor use, renal replacement therapy use, and presence of secondary AKI. Interaction terms between trajectory groups and subgroup variables were included in Cox regression models to test for effect modification, with interaction *p*‐values less than 0.05 considered statistically significant.

All statistical analyses were performed using R software version 4.3.0. Two‐sided *p*‐values less than 0.05 were considered statistically significant. Multiple testing corrections were not applied to subgroup analyses given their exploratory nature.

## 3. Results

### 3.1. Baseline Characteristics

A total of 41,295 patients diagnosed with Sepsis‐3 were identified in the MIMIC‐IV version 3.1 database. After excluding 9385 patients with non‐first hospital admissions or multiple ICU admissions, 25 patients aged 18 years or younger, 3175 patients with ICU length of stay less than 24 h, and 13,382 patients with missing WBC data within the first 7 days, 15,328 patients were included in the final study cohort (Figure 1). Among these patients, 12,846 (83.8%) survived and 2482 (16.2%) died within 28 days of ICU admission.

Baseline characteristics stratified by 28‐day mortality are presented in Table 1. Compared with survivors, non‐survivors were significantly older (72.00 [61.00, 81.00] vs. 66.00 [55.00, 76.00] years, *p*  < 0.001) and had lower body mass index (26.70 [23.01, 31.47] vs. 27.87 [24.16, 32.51] kg/m^2^, *p*  < 0.001). Non‐survivors demonstrated greater disease severity, as evidenced by higher APS III scores (58.00 [45.00, 74.00] vs. 49.00 [38.00, 62.00], *p*  < 0.001), SAPS II scores (46.00 [37.00, 55.00] vs. 39.80 [32.00, 48.00], *p*  < 0.001), SOFA scores (7.00 [4.00, 10.00] vs. 6.00 [4.00, 8.00], *p*  < 0.001), and Charlson comorbidity index (7.00 [5.00, 9.00] vs. 5.00 [3.00, 7.00], *p*  < 0.001). Regarding laboratory parameters, non‐survivors exhibited higher creatinine levels (1.30 [0.90, 2.00] vs. 1.10 [0.80, 1.76] mg/dL, *p*  < 0.001), blood urea nitrogen (29.00 [18.00, 46.60] vs. 22.00 [14.00, 36.00] mg/dL, *p*  < 0.001), and prolonged coagulation indices, including INR and PT. The incidence of secondary AKI after 7 days was significantly higher in non‐survivors compared with survivors (20.1% vs. 14.1%, *p*  < 0.001). No significant differences were observed in sex distribution, baseline WBC counts, or vasopressor use between the two groups.

**Table 1:** Baseline characteristics of sepsis patients stratified by 28‐day mortality.

Characteristics	Level (*n* = )	Survived (*n* = 12846)	Died	*p*‐Value	SMD
Age (years)		66.00 (55.00, 76.00)	72.00 (61.00, 81.00)	<0.001	0.400
Gender	Male	7450 (58.0)	1440 (58.0)	1.000	<0.001
	Female	5396 (42.0)	1042 (42.0)	—	—
Body mass index (kg/m^2^)		27.87 (24.16, 32.51)	26.70 (23.01, 31.47)	<0.001	0.157
APS III score		49.00 (38.00, 62.00)	58.00 (45.00, 74.00)	<0.001	0.456
SAPS II score		39.80 (32.00, 48.00)	46.00 (37.00, 55.00)	<0.001	0.483
OASIS score		34.00 (29.00, 40.00)	37.00 (32.00, 43.00)	<0.001	0.330
LODS score		5.60 (4.00, 7.20)	7.00 (5.00, 9.00)	<0.001	0.423
SOFA score		6.00 (4.00, 8.00)	7.00 (4.00, 10.00)	<0.001	0.305
GCS score		15.00 (13.00, 15.00)	15.00 (13.00, 15.00)	0.001	0.084
Charlson comorbidity index		5.00 (3.00, 7.00)	7.00 (5.00, 9.00)	<0.001	0.534
Heart rate (bpm		90.00 (78.00, 104.00)	91.00 (78.00, 106.00)	0.060	0.041
Mean arterial pressure (mmHg)		81.00 (71.00, 93.00)	80.00 (69.00, 93.00)	0.009	0.060
Systolic blood pressure (mmHg)		120.00 (105.00, 137.00)	119.00 (104.00, 137.00)	0.091	0.039
Diastolic blood pressure (mmHg)		66.00 (56.00, 78.00)	65.00 (55.00, 78.00)	0.104	0.038
Respiratory rate (breaths/min)		19.00 (16.00, 23.00)	21.00 (17.00, 25.00)	<0.001	0.221
SpO2 (%)		98.00 (95.00, 100.00)	97.00 (94.00, 100.00)	<0.001	0.179
Temperature (°C)		36.78 (36.44, 37.17)	36.72 (36.39, 37.06)	<0.001	0.118
White blood cell (10^9^/L)		11.90 (8.30, 16.00)	12.10 (8.60, 16.80)	0.017	0.059
Platelet count (10^9^/L)		186.00 (129.00, 251.00)	179.00 (117.00, 252.00)	0.001	0.056
Hemoglobin (g/dL)		10.40 (8.90, 12.10)	10.02 (8.50, 11.80)	<0.001	0.148
Red blood cell (10¹²/L)		3.48 (2.98, 4.04)	3.36 (2.82, 3.95)	<0.001	0.148
Hematocrit (%)		32.00 (27.50, 36.70)	31.25 (26.50, 36.00)	<0.001	0.104
INR		1.30 (1.20, 1.60)	1.40 (1.20, 1.80)	<0.001	0.259
PT (seconds)		14.70 (12.90, 17.40)	15.50 (13.40, 19.40)	<0.001	0.269
APTT (seconds)		31.50 (27.70, 37.80)	33.20 (28.60, 42.38)	<0.001	0.203
Creatinine (mg/dL)		1.10 (0.80, 1.76)	1.30 (0.90, 2.00)	<0.001	0.152
BUN (mg/dL)		22.00 (14.00, 36.00)	29.00 (18.00, 46.60)	<0.001	0.333
Lactate (mmol/L)		1.80 (1.30, 2.66)	1.96 (1.40, 2.90)	<0.001	0.147
pH		7.37 (7.31, 7.42)	7.37 (7.30, 7.42)	0.352	0.041
HCO_3_ (mEq/L)		22.00 (19.00, 25.00)	22.00 (19.00, 25.00)	0.007	0.048
Sodium (mEq/L)		138.00 (135.00, 141.00)	138.00 (134.00, 141.00)	0.594	0.019
Potassium (mEq/L)		4.10 (3.70, 4.60)	4.20 (3.74, 4.70)	<0.001	0.073
Calcium (mg/dL)		8.20 (7.70, 8.70)	8.30 (7.78, 8.80)	<0.001	0.078
Chloride (mEq/L)		104.00 (100.00, 108.00)	103.00 (98.45, 107.00)	<0.001	0.138
Glucose (mg/dL)		138.00 (111.00, 176.30)	135.00 (109.00, 178.00)	0.159	0.002
Mechanical ventilation	No	5318 (41.4)	1057 (42.6)	0.281	0.024
	Yes	7528 (58.6)	1425 (57.4)		
Renal replacement therapy	No	12335 (96.0)	2342 (94.4)	<0.001	0.078
	Yes	511 (4.0)	140 (5.6)		
Vasopressor use	No	7375 (57.4)	1387 (55.9)	0.166	0.031
	Yes	5471 (42.6)	1095 (44.1)		
Insulin use	No	8202 (63.8)	1746 (70.3)	<0.001	0.139
	Yes	4644 (36.2)	736 (29.7)		
AKI after 7 days	No	11030 (85.9)	1983 (79.9)	<0.001	0.159
	Yes	1816 (14.1)	499 (20.1)		

*Note:* Data are presented as median [interquartile range] for continuous variables and *n* (%) for categorical variables. *p*‐Values were calculated using Kruska–Wallis tests for continuous variables and chi‐squared tests or Fisher’s exact tests for categorical variables. Continuous variables are presented as median [IQR]. Categorical variables are presented as *n* (%).

Abbreviations: AKI, Acute Kidney Injury; APS III, Acute Physiology Score III; APTT, Activated Partial Thromboplastin Time; BUN, Blood Urea Nitrogen; GCS, Glasgow Coma Scale; INR, International Normalized Ratio; LODS, Logistic Organ Dysfunction System; OASIS, Oxford Acute Severity of Illness Score; PT, Prothrombin Time; SAPS II, Simplified Acute Physiology Score II; SMD, Standardized mean difference; SOFA, Sequential Organ Failure Assessment.

### 3.2. WBC Trajectory Patterns

**Figure 2:**
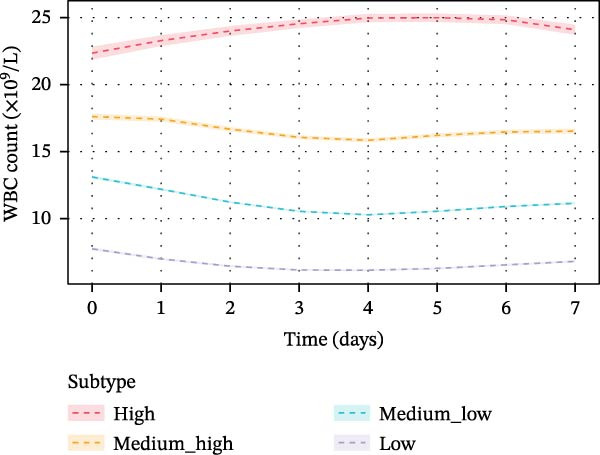
White blood cell count trajectories over the first 7 days of ICU admission identified by group‐based trajectory modeling. Four distinct trajectory groups were identified: high (red), medium–high (orange), medium–ow (cyan), and ow (purple). Lines represent group mean values, and shaded areas represent 95% confidence intervals. The *x*‐axis indicates time in days from ICU admission, and the *y*‐axis indicates white blood cell count (×10^9^/L).

Using GBTM, four distinct WBC trajectory patterns were identified over the first 7 days of ICU admission (Figure 2). The trajectory groups were designated as high, medium–high, medium–low, and low based on their overall mean WBC counts of 24.13, 16.60, 11.24, and 6.64 × 10^9^/L, respectively. The High trajectory group comprised 812 patients (5.3%), characterized by elevated initial WBC counts (22.35 ± 6.91 × 10^9^/L on day 0) that peaked on day 5 (24.99 ± 4.49 × 10^9^/L) before slightly declining. The medium–high group included 2,830 patients (18.5%), demonstrating initial counts of 17.62 ± 5.87 × 10^9^/L with a gradual decrease to 15.85 ± 3.65 × 10^9^/L by day 4, followed by mild recovery. The medium–low group was the largest subgroup with 7,121 patients (46.5%), showing initial values of 13.10 ± 4.48 × 10^9^/L that declined to 10.28 ± 2.53 × 10^9^/L by day 4 before stabilizing. The low trajectory group lconsisted of 4,565 patients (29.8%), presenting with the lowest initial counts (7.75 ± 3.40 × 10^9^/L) that further decreased to 6.15 ± 2.20 × 10^9^/L by day 4. The clustering quality was assessed by silhouette coefficient (0.46), Calinski–Harabasz index (9161.2), and Davies–Bouldin index (1.22), indicating adequate separation between trajectory groups.

### 3.3. Association Between WBC Trajectories and Mortality Outcomes

The association between WBC trajectory groups and mortality outcomes was further evaluated using Kaplan–Meier survival analysis and Cox proportional hazards regression (Figure 3). In the overall cohort, significant differences in 28‐day mortality were observed across trajectory groups (log‐rank *p*  < 0.001, Figure 3A). The High trajectory group exhibited the highest 28‐day mortality rate (31.2%, 253/812), followed by medium–high (20.7%, 585/2830), medium–low (14.8%, 1057/7121), and low (12.9%, 587/4565). Using the high‐trajectory group as reference, Cox regression demonstrated significantly reduced mortality risk in other groups: medium–high (HR: 0.62, 95% CI: 0.53–0.71, *p*  < 0.001), medium–low (HR: 0.43, 95% CI: 0.37–0.49, *p*  < 0.001), and low (HR: 0.36, 95% CI: 0.31–0.42, *p*  < 0.001). Similar patterns were observed for 90‐day mortality (log‐rank *p*  < 0.001, Figure 3B), with mortality rates of 45.3%, 31.5%, 24.8%, and 23.6% for the high, edium–high, medium–low, and low groups, respectively.

Figure 3Kaplan–Meier survival curves for mortality outcomes stratified by white blood cell trajectory groups. (A) 28‐day mortality in the overall cohort. (B) 90‐day mortality in the overall cohort. (C) 28‐day mortality in patients with secondary acute kidney injury. (D) 90‐day mortality in patients with secondary acute kidney injury. *p*‐Values were calculated using log‐rank tests. Trajectory groups are color‐coded as: high (red), medium–high (orange), medium–low (cyan), and low (purple).

**Figure:**
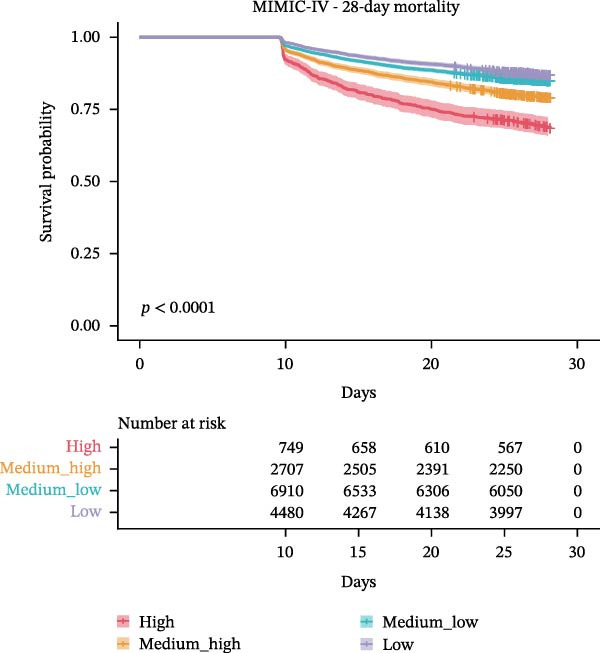
(A)

**Figure:**
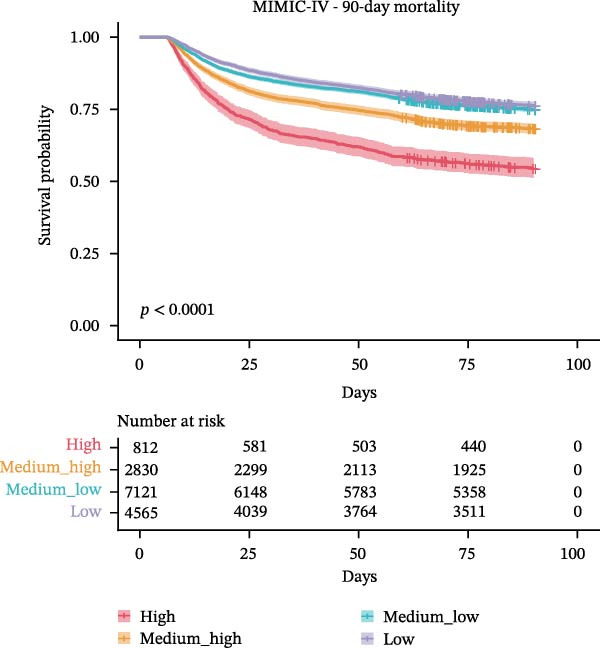
(B)

**Figure:**
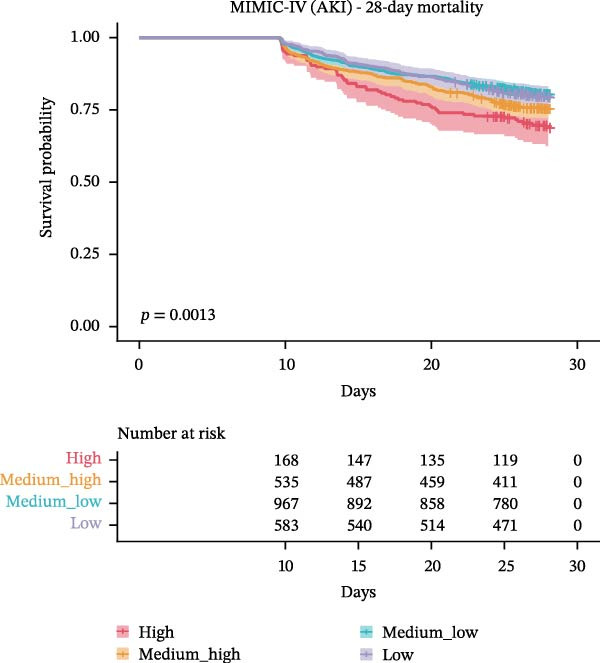
(C)

**Figure:**
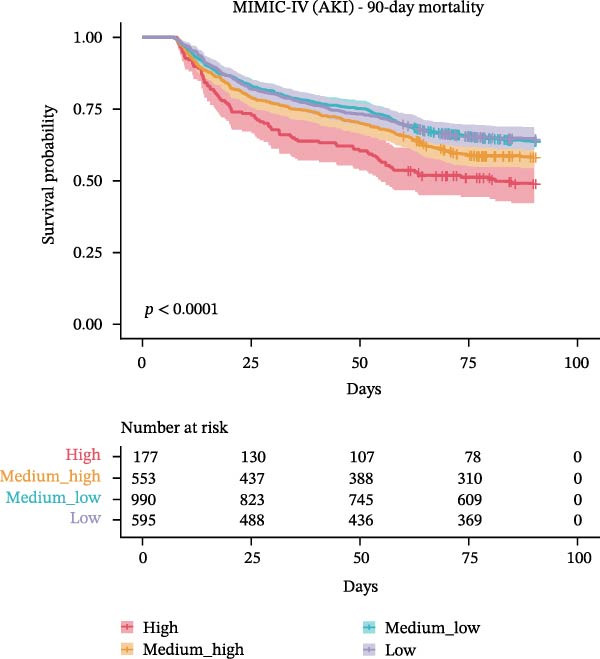
(D)

Subgroup analysis in patients with secondary AKI (*n* = 2,315) revealed consistent findings. The 28‐day mortality rates were 30.5%, 24.2%, 19.2%, and 20.3% for the high, medium–high, medium–low, and low trajectory groups, respectively (log‐rank *p* = 0.001, Figure 3C). Compared with the high group, the medium–low (HR: 0.58, 95% CI: 0.43–0.79, *p*  < 0.001), and low (HR: 0.61, 95% CI: 0.44–0.84, *p* = 0.003) groups demonstrated significantly lower 28‐day mortality risk, while the medium–high group showed a nonsignificant trend toward reduced risk (HR: 0.76, 95% CI: 0.55–1.04, *p* = 0.082). For 90‐day mortality in AKI patients (Figure 3D), all trajectory groups showed significantly lower mortality risk compared with the high group: medium–high (HR: 0.74, 95% CI: 0.58–0.95, *p* = 0.016), medium–low (HR: 0.61, 95% CI: 0.49–0.77, *p*  < 0.001), and low (HR: 0.60, 95% CI: 0.47–0.77, *p*  < 0.001).

### 3.4. Clinical Characteristics Across Trajectory Groups

**Figure 4:**
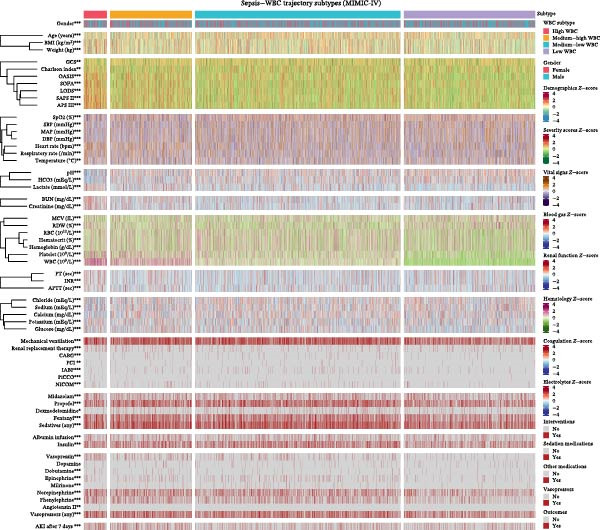
Heatmap of baseline clinical characteristics across white blood cell trajectory groups. Continuous variables are presented as *z*‐scores, with red indicating higher values and blue indicating lower values. Binary variables are presented with red indicating presence and gray indicating absence. Variables are grouped by category: demographics, severity scores, vital signs, blood gas parameters, renal function, hematology, coagulation, electrolytes, interventions, medications, and outcomes. Asterisks indicate statistical significance:  ^∗^ *p*  < 0.05,  ^∗∗^ *p*  < 0.01,  ^∗∗∗^ *p*  < 0.001.

To further characterize the identified trajectory groups, baseline clinical characteristics were compared across the four WBC trajectory patterns (Figure 4, Table 2). Significant differences were observed in demographic characteristics, with the high‐trajectory group having a lower proportion of male patients (53.8%) compared with the low group (61.0%, *p*  < 0.001), younger age (62.36 ± 16.01 vs. 65.16 ± 15.57 years), and higher body mass index (29.47 ± 7.03 vs. 27.99 ± 6.44 kg/m^2^). Notably, patients in the high‐trajectory group demonstrated significantly greater disease severity, as evidenced by higher APS III scores (67.09 ± 20.91 vs. 50.86 ± 18.29, *p*  < 0.001), SAPS II scores (48.91 ± 14.14 vs. 39.96 ± 12.53, *p*  < 0.001), and SOFA scores (7.94 ± 3.96 vs. 6.33 ± 3.29, *p*  < 0.001) compared with the low group.

**Table 2:** Comparison of baseline clinical characteristics across white blood cell trajectory groups.

Feature	Subtype_1	Subtype_2	Subtype_3	Subtype_4	*p*‐Value
Gender	437/812 (53.8%)	1547/2830 (54.7%)	4121/7121 (57.9%)	2785/4565 (61.0%)	<0.001
Age (years)	62.36 ± 16.01	64.33 ± 15.90	65.94 ± 15.68	65.16 ± 15.57	<0.001
Weight (kg)	84.72 ± 22.80	83.47 ± 22.06	82.89 ± 21.50	80.80 ± 21.07	<0.001
BMI (kg/m²)	29.47 ± 7.03	29.17 ± 6.90	28.82 ± 6.61	27.99 ± 6.44	<0.001
APS III	67.09 ± 20.91	57.02 ± 20.70	50.90 ± 19.30	50.86 ± 18.29	<0.001
SAPS II	48.91 ± 14.14	43.79 ± 13.81	40.98 ± 12.89	39.96 ± 12.53	<0.001
OASIS	38.48 ± 8.62	36.00 ± 8.25	35.00 ± 7.80	33.82 ± 7.70	<0.001
LODS	7.32 ± 2.96	6.40 ± 2.89	5.78 ± 2.73	5.63 ± 2.67	<0.001
SOFA	7.94 ± 3.96	6.76 ± 3.63	6.06 ± 3.35	6.33 ± 3.29	<0.001
GCS	13.61 ± 2.35	13.62 ± 2.38	13.46 ± 2.55	13.43 ± 2.54	0.009
Charlson Index	5.50 ± 3.03	5.32 ± 2.90	5.44 ± 2.89	5.59 ± 2.88	0.002
MAP (mmHg)	80.20 ± 17.05	81.81 ± 17.23	83.56 ± 17.73	82.98 ± 17.62	<0.001
SBP (mmHg)	117.25 ± 22.74	120.13 ± 23.65	122.42 ± 24.13	122.68 ± 23.96	<0.001
DBP (mmHg)	65.88 ± 16.43	66.56 ± 16.70	68.05 ± 17.08	68.05 ± 16.98	<0.001
Heart rate (bpm)	99.16 ± 20.65	95.01 ± 19.93	90.98 ± 19.86	89.90 ± 20.24	<0.001
Temperature (°C)	36.83 ± 0.70	36.80 ± 0.74	36.78 ± 0.73	36.82 ± 0.76	0.006
Respiratory Rate (/min)	22.29 ± 6.27	20.77 ± 5.82	19.90 ± 5.60	19.83 ± 5.61	<0.001
SpO2 (%)	96.11 ± 3.38	96.66 ± 3.34	97.08 ± 3.20	97.17 ± 3.10	<0.001
pH	7.33 ± 0.10	7.35 ± 0.09	7.36 ± 0.09	7.37 ± 0.08	<0.001
HCO3 (mEq/L)	20.26 ± 4.95	21.51 ± 4.62	22.22 ± 4.45	22.57 ± 4.70	<0.001
Lactate (mmol/L)	2.64 ± 1.57	2.43 ± 1.49	2.22 ± 1.34	2.08 ± 1.28	<0.001
Creatinine (mg/dL)	1.97 ± 1.34	1.59 ± 1.16	1.45 ± 1.06	1.50 ± 1.15	<0.001
BUN (mg/dL)	35.54 ± 20.90	30.80 ± 20.11	27.79 ± 18.69	28.62 ± 19.81	<0.001
WBC (10^9^/L)	20.40 ± 8.12	17.12 ± 6.67	13.24 ± 5.10	8.27 ± 3.90	<0.001
RBC (10^12^/L)	3.46 ± 0.80	3.55 ± 0.79	3.58 ± 0.77	3.37 ± 0.76	<0.001
Hematocrit (%)	31.55 ± 6.82	32.27 ± 6.86	32.80 ± 6.73	31.04 ± 6.57	<0.001
RDW (%)	15.87 ± 2.27	15.29 ± 2.09	15.00 ± 1.98	15.60 ± 2.16	<0.001
Hemoglobin (g/dL)	10.25 ± 2.25	10.53 ± 2.30	10.75 ± 2.27	10.17 ± 2.20	<0.001
Platelet (10^9^/L)	229.95 ± 117.25	225.69 ± 105.47	204.46 ± 91.42	158.38 ± 85.28	<0.001
MCV (fL)	91.67 ± 7.27	91.45 ± 6.93	91.64 ± 6.71	92.55 ± 7.24	<0.001
INR	1.60 ± 0.48	1.51 ± 0.48	1.45 ± 0.46	1.50 ± 0.50	<0.001
PT (sec)	17.45 ± 4.97	16.41 ± 4.86	15.84 ± 4.66	16.22 ± 5.05	<0.001
APTT (sec)	37.90 ± 13.70	35.61 ± 12.82	35.15 ± 13.03	35.34 ± 11.93	<0.001
Calcium (mg/dL)	8.01 ± 0.84	8.18 ± 0.79	8.24 ± 0.77	8.22 ± 0.80	<0.001
Chloride (mEq/L)	102.36 ± 7.22	103.27 ± 6.38	103.68 ± 6.09	103.51 ± 6.27	<0.001
Sodium (mEq/L)	136.69 ± 5.47	137.36 ± 4.95	137.73 ± 4.69	137.86 ± 4.86	<0.001
Potassium (mEq/L)	4.27 ± 0.80	4.24 ± 0.73	4.22 ± 0.74	4.18 ± 0.73	<0.001
Glucose (mg/dL)	152.95 ± 60.74	155.46 ± 59.72	154.58 ± 59.39	144.15 ± 57.48	<0.001
Mechanical ventilation	504/812 (62.1%)	1743/2830 (61.6%)	4393/7121 (61.7%)	2313/4565 (50.7%)	<0.001
Renal replacement therapy	105/812 (12.9%)	166/2830 (5.9%)	211/7121 (3.0%)	169/4565 (3.7%)	<0.001
CABG	4/812 (0.5%)	60/2830 (2.1%)	171/7121 (2.4%)	52/4565 (1.1%)	<0.001
PCI	6/812 (0.7%)	24/2830 (0.8%)	56/7121 (0.8%)	13/4565 (0.3%)	0.004
IABP	13/812 (1.6%)	67/2830 (2.4%)	190/7121 (2.7%)	60/4565 (1.3%)	<0.001
PiCCO	11/812 (1.4%)	12/2830 (0.4%)	25/7121 (0.4%)	7/4565 (0.2%)	<0.001
NICOM	47/812 (5.8%)	108/2830 (3.8%)	152/7121 (2.1%)	75/4565 (1.6%)	<0.001
Midazolam	205/812 (25.2%)	556/2830 (19.6%)	1352/7121 (19.0%)	841/4565 (18.4%)	<0.001
Propofol	326/812 (40.1%)	1296/2830 (45.8%)	3389/7121 (47.6%)	1709/4565 (37.4%)	<0.001
Dexmedetomidine	32/812 (3.9%)	160/2830 (5.7%)	449/7121 (6.3%)	239/4565 (5.2%)	0.010
Fentanyl	389/812 (47.9%)	1284/2830 (45.4%)	2974/7121 (41.8%)	1612/4565 (35.3%)	<0.001
Sedatives (any)	454/812 (55.9%)	1663/2830 (58.8%)	4177/7121 (58.7%)	2226/4565 (48.8%)	<0.001
Albumin infusion	135/812 (16.6%)	480/2830 (17.0%)	1078/7121 (15.1%)	562/4565 (12.3%)	<0.001
Insulin	276/812 (34.0%)	1064/2830 (37.6%)	2685/7121 (37.7%)	1355/4565 (29.7%)	<0.001
Vasopressin	159/812 (19.6%)	334/2830 (11.8%)	490/7121 (6.9%)	187/4565 (4.1%)	<0.001
Dopamine	23/812 (2.8%)	80/2830 (2.8%)	205/7121 (2.9%)	108/4565 (2.4%)	0.390
Dobutamine	21/812 (2.6%)	65/2830 (2.3%)	98/7121 (1.4%)	50/4565 (1.1%)	<0.001
Epinephrine	48/812 (5.9%)	179/2830 (6.3%)	415/7121 (5.8%)	103/4565 (2.3%)	<0.001
Milrinone	9/812 (1.1%)	55/2830 (1.9%)	169/7121 (2.4%)	42/4565 (0.9%)	<0.001
Norepinephrine	317/812 (39.0%)	959/2830 (33.9%)	1977/7121 (27.8%)	1080/4565 (23.7%)	<0.001
Phenylephrine	193/812 (23.8%)	657/2830 (23.2%)	1552/7121 (21.8%)	689/4565 (15.1%)	<0.001
Angiotensin II	3/812 (0.4%)	2/2830 (0.1%)	1/7121 (0.0%)	1/4565 (0.0%)	0.004
Vasopressors (any)	395/812 (48.6%)	1352/2830 (47.8%)	3168/7121 (44.5%)	1651/4565 (36.2%)	<0.001
AKI after 7 days	177/812 (21.8%)	553/2830 (19.5%)	990/7121 (13.9%)	595/4565 (13.0%)	<0.001

*Note:* Continuous variables are presented as mean ± standard deviation, and categorical variables are presented as n/N (%). *p*‐Values were calculated using Kruskal–Wallis tests for continuous variables and chi‐squared tests or Fisher’s exact tests for categorical variables.

Regarding physiological parameters, the high‐trajectory group exhibited more pronounced hemodynamic instability with lower mean arterial pressure (80.20 ± 17.05 vs. 82.98 ± 17.62 mmHg, *p*  < 0.001), higher heart rate (99.16 ± 20.65 vs. 89.90 ± 20.24 bpm, *p*  < 0.001), and elevated respiratory rate (22.29 ± 6.27 vs. 19.83 ± 5.61 breaths/min, *p*  < 0.001). Laboratory parameters revealed that patients in the high group had lower arterial pH (7.33 ± 0.10 vs. 7.37 ± 0.08, *p*  < 0.001), higher lactate levels (2.64 ± 1.57 vs. 2.08 ± 1.28 mmol/L, *p*  < 0.001), elevated creatinine (1.97 ± 1.34 vs. 1.50 ± 1.15 mg/dL, *p*  < 0.001), and prolonged coagulation indices including INR (1.60 ± 0.48 vs. 1.50 ± 0.50, *p*  < 0.001), and APTT (37.90 ± 13.70 vs. 35.34 ± 11.93 s, *p*  < 0.001). The high‐trajectory group also required more intensive interventions, including higher rates of renal replacement therapy (12.9% vs. 3.7%, *p*  < 0.001), vasopressor use (48.6% vs. 36.2%, *p*  < 0.001), and norepinephrine administration (39.0% vs. 23.7%, *p*  < 0.001). Furthermore, the incidence of secondary AKI after 7 days was significantly higher in the high‐trajectory group compared with the low group (21.8% vs. 13.0%, *p*  < 0.001).

### 3.5. Subgroup Analyses

**Figure 5:**
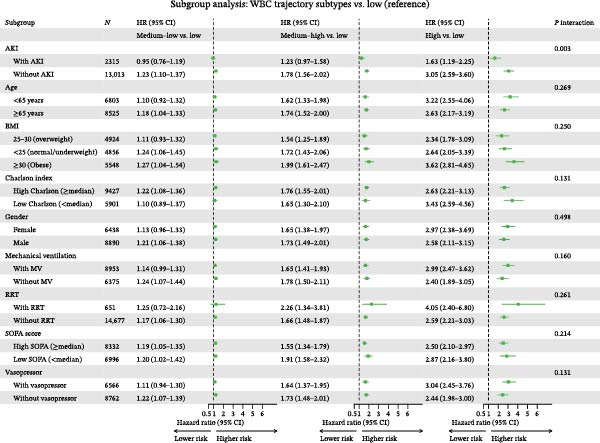
Forest plot of subgroup analyses for the association between white blood cell trajectory groups and 28‐day mortality. Hazard ratios with 95% confidence intervals are shown for the high‐trajectory group compared with the low‐trajectory group (reference) within each subgroup. *p*‐values for interaction test the heterogeneity of effect across subgroup levels. Abbreviations: AKI, Acute Kidney Injury; BMI, Body Mass Index; CI, Confidence Interval; HR, Hazard Ratio; MV, Mechanical Ventilation; RRT, Renal Replacement Therapy; SOFA, Sequential Organ Failure Assessment.

Subgroup analyses were performed to evaluate the consistency of the association between WBC trajectory groups and 28‐day mortality across clinically relevant patient characteristics (Figure 5). The elevated mortality risk associated with the High trajectory group compared with the Low group remained consistent across all pre‐specified subgroups, including sex (male: HR: 2.58, 95% CI: 2.11–3.15; female: HR: 2.97, 95% CI: 2.38–3.69), age (<65 years: HR: 3.22, 95% CI: 2.55–4.06; ≥65 years: HR; 2.63, 95% CI: 2.17–3.19), BMI categories (normal/underweight: HR: 2.64, 95% CI: 2.05–3.39; overweight: HR: 2.34, 95% CI: 1.78–3.09; obese: HR: 3.62, 95% CI: 2.81–4.65), SOFA score (low: HR: 2.87, 95% CI: 2.16–3.80; high: HR; 2.50, 95% CI: 2.10–2.97), Charlson comorbidity index (low: HR: 3.43, 95% CI: 2.59–4.56; high: HR: 2.63, 95% CI: 2.21–3.13), mechanical ventilation status (with: HR: 2.99, 95% CI: 2.47–3.62; without: HR: 2.40, 95% CI: 1.89–3.05), vasopressor use (with: HR: 3.04, 95% CI: 2.45–3.76; without: HR: 2.44, 95% CI; 1.98–3.00), and renal replacement therapy (with: HR: 4.05, 95% CI: 2.40–6.80; without: HR: 2.59, 95% CI: 2.21–3.03). Notably, a significant interaction was observed for AKI status (*P* for interaction = 0.003), with the high‐trajectory group showing a stronger association with mortality in patients without AKI (HR: 3.05, 95% CI: 2.59–3.60) compared with those with AKI (HR: 1.63, 95% CI: 1.19–2.25). No significant interactions were detected for other subgroup variables (all *P* for interaction > 0.05).

## 4. Discussion

In this retrospective cohort study of 15,328 sepsis patients from the MIMIC‐IV database, we identified four distinct WBC trajectory patterns during the first 7 days of ICU admission using GBTM. The High trajectory group, characterized by persistently elevated WBC counts (overall mean 24.13 × 10^9^/L), demonstrated the highest 28‐day mortality rate (31.2%) and was independently associated with increased mortality risk compared with the Low trajectory group (HR: 0.36, 95% CI: 0.31–0.42 for low vs. high). Secondary findings revealed that patients in the High trajectory group exhibited greater disease severity, more pronounced hemodynamic instability, and higher rates of secondary AKI after 7 days (21.8% vs. 13.0%). Subgroup analyses demonstrated consistent associations across most clinically relevant patient characteristics, with a significant interaction observed for AKI status, where the High trajectory showed stronger mortality associations in patients without AKI compared to those with AKI.

Our findings align with and extend previous research demonstrating the prognostic significance of WBC dynamics in sepsis. Rimmer et al. identified seven distinct WBC trajectories in 917 patients with septic shock, reporting that a rising WBC trajectory was independently associated with increased 30‐day mortality (HR: 3.41, 95% CI: 1.86–6.26) compared with stable trajectories [6]. Similarly, Miao et al. analyzed 7,410 sepsis patients using latent class mixed models and identified eight leukocyte trajectory subtypes, finding that patients with persistently high WBC levels had the poorest prognosis (HR: 3.00, 95% CI: 2.48–3.62) [7]. Our study corroborates these findings in a larger cohort and provides additional granularity by characterizing four clinically interpretable trajectory patterns with distinct baseline characteristics and outcome profiles. The consistency of these associations across different databases and analytical approaches strengthens the evidence that longitudinal WBC patterns offer superior prognostic information compared to single‐timepoint measurements.

The observed association between high WBC trajectories and adverse outcomes likely reflects the complex interplay between inflammation, immune dysfunction, and organ damage in sepsis. Elevated and persistent leukocytosis may indicate an exaggerated inflammatory response that contributes to tissue injury through mechanisms including microcirculatory disturbances, metabolic reprograming, and programed cell death pathways such as pyroptosis [11, 12]. Biomarker studies have demonstrated that WBC‐derived inflammatory indices, including neutrophil‐to‐lymphocyte ratio and systemic immune‐inflammation index, are closely associated with mortality in SA‐AKI patients [5]. Furthermore, the pathophysiological mechanisms underlying SA‐AKI involve not only hemodynamic alterations but also direct inflammatory injury to renal tubular cells and endothelial dysfunction [13]. Our finding that the high‐trajectory group required more intensive interventions, including higher rates of renal replacement therapy (12.9% vs. 3.7%) and vasopressor use (48.6% vs. 36.2%), supports the concept that persistent hyperinflammation drives multi‐organ dysfunction and necessitates aggressive supportive care.

Interestingly, our subgroup analysis revealed a significant interaction between WBC trajectory and AKI status (*P* for interaction = 0.003), with attenuated mortality associations observed in patients with secondary AKI. This finding may reflect the complex immunological milieu in AKI patients, where both hyperinflammatory and hypoinflammatory states can contribute to adverse outcomes [8]. Li et al. [8] demonstrated that lymphocyte trajectory phenotypes in sepsis patients were associated with divergent outcomes, with the high‐declining phenotype exhibiting the highest mortality (25.9%). The differential response patterns between AKI and non‐AKI patients suggest that the prognostic value of WBC trajectories may be modified by the presence of organ dysfunction, potentially reflecting differences in immune cell trafficking, sequestration, or functional impairment in the setting of renal injury. These observations underscore the importance of considering organ‐specific factors when interpreting inflammatory biomarkers in sepsis.

The epidemiological context of our findings is consistent with contemporary studies on SA‐AKI. White et al. reported that SA‐AKI occurred in approximately one in six ICU patients and was diagnosed predominantly on the first day of admission [14]. Takeuchi et al. found that SA‐AKI was present in nearly half of sepsis patients and was associated with increased hospital mortality (adjusted HR: 1.59, 95% CI: 1.51–1.66) [2]. Song et al. [15] demonstrated that 62.3% of sepsis patients developed SA‐AKI, with severe stages associated with significantly increased mortality risk. Our observation that the high‐trajectory group had a 21.8% incidence of secondary AKI compared to 13.0% in the low group aligns with these findings and suggests that persistent hyperinflammation may predispose patients to delayed renal complications. The clinical implications of this association warrant further investigation, as secondary AKI occurring after the initial resuscitation phase may represent a distinct pathophysiological entity with different management requirements [16].

Several methodological considerations and potential confounders warrant discussion. Machine learning‐based subphenotyping approaches have demonstrated utility in identifying clinically meaningful sepsis subtypes with distinct characteristics and outcomes [9]. Hu et al. [9] identified two sepsis subphenotypes using K‐means clustering, with subphenotype B (characterized by higher WBC counts) showing significantly higher in‐hospital mortality (29.4% vs. 8.5%). Lai et al. [17] employed unsupervised consensus clustering to identify subphenotypes in dialysis‐requiring SA‐AKI, discovering that hyperlactatemia ≥3.3 mmol/L was an independent outcome predictor. These complementary approaches highlight the heterogeneity of sepsis and the potential for trajectory‐based analyses to inform precision medicine strategies. However, unmeasured confounders such as antibiotic timing, fluid resuscitation volume, and WBC differential counts (neutrophils and lymphocytes) may influence the observed associations and should be considered when interpreting our results [4].

This study has several strengths. First, we utilized a large, well‐characterized cohort from the MIMIC‐IV database with comprehensive longitudinal data, enabling robust trajectory modeling. Second, our analytical approach using GBTM allowed identification of clinically interpretable trajectory patterns without imposing arbitrary cutoffs. Third, we performed extensive subgroup analyses to evaluate the consistency of associations across patient characteristics, providing evidence for the generalizability of our findings. Fourth, we examined multiple clinically relevant outcomes, including mortality, AKI, and the need for intensive interventions. However, several limitations must be acknowledged. The retrospective, single‐center design limits causal inference and generalizability to other populations, including non‐Western healthcare settings and community hospitals. Daily WBC counts were obtained as the first available value within each 24 h period, and multiple measurements were averaged when available; however, diurnal variations and the influence of therapeutic interventions could not be fully controlled. Certain potential confounders, including antibiotic timing, specific pathogen identification, and WBC subset counts, were not available for adjustment. Additionally, our analysis was restricted to complete 24 h datasets, potentially introducing selection bias by excluding patients with missing data.

Our findings have important clinical implications for sepsis management. The identification of distinct WBC trajectory patterns provides a framework for early risk stratification that could guide clinical decision‐making regarding monitoring intensity, therapeutic interventions, and resource allocation. Patients exhibiting high WBC trajectories may benefit from enhanced surveillance for multi‐organ dysfunction and consideration of immunomodulatory therapies, although prospective validation is required before clinical implementation. The significant interaction with AKI status suggests that personalized approaches accounting for organ‐specific factors may improve prognostic accuracy. Future research should focus on external validation of these trajectory patterns in diverse populations, integration of WBC trajectories with other biomarkers and clinical scoring systems, and prospective evaluation of trajectory‐guided therapeutic strategies. Investigation of the mechanistic links between WBC dynamics and organ injury, including the role of specific leukocyte subsets and their activation states, may identify novel therapeutic targets for SA‐AKI prevention and treatment [18].

## 5. Conclusions

In this large retrospective cohort of sepsis patients, GBTM identified four distinct WBC trajectory patterns over the first 7 days of ICU admission, with the high‐trajectory group demonstrating significantly increased 28‐day and 90‐day mortality, greater disease severity, and higher incidence of secondary AKI compared with lower trajectory groups. These associations remained consistent across most clinically relevant subgroups, with a notable interaction observed for AKI status. The trajectory classification showed adequate separation quality as assessed by silhouette coefficient, Calinski–Harabasz index, and Davies–Bouldin index. Our findings suggest that longitudinal WBC trajectory patterns provide prognostic information beyond single‐timepoint measurements and may serve as a practical tool for early risk stratification in sepsis patients. Future prospective studies are warranted to validate these trajectory patterns in external cohorts and to evaluate their potential utility in guiding personalized therapeutic interventions.

## Author Contributions

**Jie Yan**: conceptualization, methodology, software, formal analysis, data curation, visualization, writing – original draft. **Ailifeire Abudurexiti**: methodology, validation, investigation, writing – review and editing. **Guligeina Yibubula**: resources, supervision, project administration, funding acquisition. **Wencai Li**: validation, writing – review and editing. **Jing Liang**: supervision, project administration, funding acquisition, writing – review and editing.

## Funding

The authors’ project funding comes from The Xinjiang Uygur Autonomous Region Health and Wellness Science and Technology Plan Project (Project Number 2025001CXKYXM650027150).

## Disclosure

All authors read and approved the final manuscript.

## Ethics Statement

This study utilized the publicly available, de‐identified MIMIC‐IV database (Version 3.1). The creation and maintenance of MIMIC‐IV were approved by the Institutional Review Board (IRB) of the Massachusetts Institute of Technology (MIT), which granted a waiver of the informed consent requirement due to the retrospective use of fully de‐identified clinical data. Therefore, no additional ethical approval was required for this secondary analysis.

## Consent

As this study exclusively used de‐identified retrospective data from MIMIC‐IV, no individual patient consent for publication was applicable or required.

## Conflicts of Interest

The authors declare no conflicts of interest.

## Data Availability

The datasets analyzed in this study are available in the MIMIC‐IV repository on PhysioNet: https://physionet.org/content/mimiciv/. Access requires completion of PhysioNet’s credentialing process (including CITI training).
